# 3D bioprinting: improving *in vitro* models of metastasis with heterogeneous tumor microenvironments

**DOI:** 10.1242/dmm.025049

**Published:** 2017-01-01

**Authors:** Jacob L. Albritton, Jordan S. Miller

**Affiliations:** Department of Bioengineering, Rice University, Houston, TX 77005, USA

**Keywords:** 3D bioprinting, Metastasis, *In vitro* model, Tumor microenvironment, Cancer

## Abstract

Even with many advances in treatment over the past decades, cancer still remains a leading cause of death worldwide. Despite the recognized relationship between metastasis and increased mortality rate, surprisingly little is known about the exact mechanism of metastatic progression. Currently available *in vitro* models cannot replicate the three-dimensionality and heterogeneity of the tumor microenvironment sufficiently to recapitulate many of the known characteristics of tumors *in vivo*. Our understanding of metastatic progression would thus be boosted by the development of *in vitro* models that could more completely capture the salient features of cancer biology. Bioengineering groups have been working for over two decades to create *in vitro* microenvironments for application in regenerative medicine and tissue engineering. Over this time, advances in 3D printing technology and biomaterials research have jointly led to the creation of 3D bioprinting, which has improved our ability to develop *in vitro* models with complexity approaching that of the *in vivo* tumor microenvironment. In this Review, we give an overview of 3D bioprinting methods developed for tissue engineering, which can be directly applied to constructing *in vitro* models of heterogeneous tumor microenvironments. We discuss considerations and limitations associated with 3D printing and highlight how these advances could be harnessed to better model metastasis and potentially guide the development of anti-cancer strategies.

## Abstract (precis)

**Summary:** Here, Albritton and Miller discuss recent 3D bioprinting advances that can be used to produce heterogeneous tumor microenvironments for improved physical modeling of cancer metastasis.

## Introduction

Despite substantial progress in cancer research over the past century, the World Health Organization (WHO) reported over eight million cancer-related deaths worldwide in 2012, with complications from metastases given as the major cause of death (Torre et al., 2015). Metastatic spread, in which cancerous cells spread from a primary tumor to distant and distinct tissues, amplifies the impact of metastasis on affected individuals. Metastatic spread is also associated with a significant decrease in 5-year survival rates (Siegel et al., 2015) and is linked to as many as 90% of deaths from cancer (Chaffer and Weinberg, 2011). Although the relationship between metastasis and increased cancer mortality is well established, much of our knowledge about cancer has focused on advanced-stage disease, because malignant progression in humans can be asymptomatic and span decades. A better understanding of the biophysical and biochemical environments in which cancer cells operate during disease progression could enable elucidation of the underlying mechanisms of cancer progression, thereby improving identification of therapeutic targets (Jain, 2013; Quail and Joyce, 2013).

**Fig. 1.**
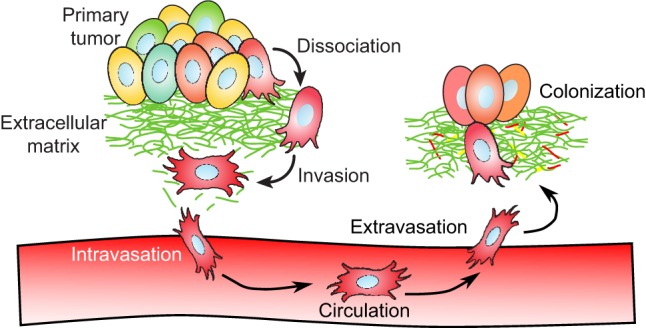
**Progression of events during metastatic disease.** Cancer cells follow a series of steps during the course of metastatic disease, potentially invading as individuals or as clusters of cells. At the start of metastatic progression, tumor cells dissociate and locally invade tissue surrounding a primary tumor. Invasive tumor cells can eventually intravasate across the endothelial barrier and circulate through the bloodstream. Rarely, a small subset of circulating tumor cells will extravasate back across the endothelial barrier into distant tissue. At these secondary sites, another small subset of colonies will further adapt to the new secondary site and proliferate to form new macroscopic tumor sites.

The change from a single primary tumor to multifocal disease follows a progression of events during which cancer cells disperse from the primary tumor site and colonize tissue at distant locations (Fig. 1) (Chaffer and Weinberg, 2011; Chambers et al., 2002; Massagué and Obenauf, 2016). Because cancer is multifaceted and encompasses a group of diseases, mechanisms through which tumor cells can spread from the primary tumor are highly varied. In one mechanism, cancer cells degrade their surrounding extracellular matrix (ECM) and actively invade surrounding tissue either as individuals or as clusters of cells (Chambers et al., 2002; Friedl and Wolf, 2003; Haeger et al., 2015). Alternatively, tumor cells can initiate uncontrolled angiogenic signaling to form underdeveloped capillaries that both supply tumors with nutrients for increased proliferation and facilitate tumor cell intravasation (Box 1) into the bloodstream (Chambers et al., 2002). Subsets of circulating tumor cells can extravasate into secondary tissue sites, and eventually some of these secondary colonies can form tumors and recruit blood vessels to establish a new tumor (Chaffer and Weinberg, 2011; Massagué and Obenauf, 2016).

**Box 1. Glossary**

**Intravasation:** During metastasis, refers to the process of cancer cells moving across the endothelial barrier into the bloodstream.

**Extravasation:** During metastasis, refers to cancer cell exit from the blood stream across the endothelial barrier.

**Colonization:** In the context of metastasis, this refers to cancer cells taking residence at a site distant from the tumor site of origin.

**Tumor microenvironment:** Local environmental conditions immediately surrounding a tumor, including the extracellular matrix, neighboring stromal cells, soluble growth factors, blood vessels, nutrients and waste.

**Heterogeneity:** Composed of or including more than one component. In the context of the tumor microenvironment, heterogeneity refers to the diverse composition of the local environment surrounding the tumor.

**Three-dimensional printing (3DP):** The act of constructing a 3D patterned object from physical material using an automated machine; analogous to a standard electronic printer that constructs 2D patterns using ink.

**Biomaterial:** Synthetic or natural material that is compatible with living tissue or cells.

**Bioink:** Mixture used for 3D printing that is composed of some combination of matrix-like scaffold biomaterial, cells or bioactive additives.

**Layer-by-layer:** Refers to the construction of a 3D object by iteratively stacking layers of material.

**Extrusion:** The process of material being physically forced through an opening. In 3D printing, this refers to pressurized, controlled ejection of material through a small nozzle.

**Inkjet printing:** In 3D printing, this refers to the use of a specialized nozzle capable of rapidly ejecting controlled volumes of material in the form of a droplet.

**Photopolymerization:** Refers to a light-initiated polymerization cross-linking reaction. In the context of 3D printing, this reaction typically converts illuminated liquid material into a solid phase.

**Projection stereolithography:** Use of a patterned light source, such as a commercial light projector, to cure light-sensitive material.

**Laser sintering:** Use of a focused laser beam to fuse granules of a powdered material with heat.

**Sacrificial casting:** A technique for creating 3D objects by casting material around a mold, then selectively removing the original mold through physical removal or chemical dissolution.

**Fig. 2.**
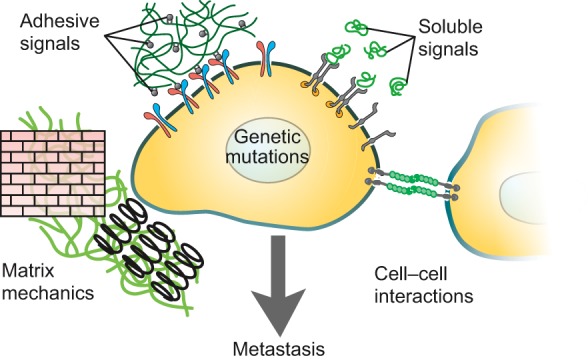
**Tumor microenvironment features that affect metastatic progression.** Features of the tumor microenvironment are thought to play a role in facilitating or promoting tumorigenic behavior. These features include adhesive signals from extracellular matrix components such as collagen and fibrin; soluble signals like growth factors and cytokines; extracellular matrix mechanical features including stiffness and local tension or compression; and cell–cell interactions with intra- and extra-tumoral stromal cells. Adapted with permission from Hubbell (2008) and Lutolf and Hubbell (2005).

According to the Somatic Mutation Theory of cancer, the acquisition of invasive traits is largely attributed to genetic mutation followed by Darwinian selection of ‘fit’ tumor cells (Greaves and Maley, 2012; Grove and Vassiliou, 2014; Nowell, 1976). The acquisition of direct mutations in the DNA, phenotype switching via epigenetic changes, and chromosomal rearrangements or duplications have all been observed to influence and promote tumor proliferation and migration (Greaves and Maley, 2012; Merlo et al., 2006). However, a primary tumor is not just a large cluster of sub-clonal populations of cancer cells. Within this survival-of-the-fittest conceptual framework, the environmental pressures on cells should be considered and investigated on the microscale. Increasing evidence has pointed to the tumor microenvironment (TME) (Box 1) as another major driver of tumorigenic behavior on par with genetic mutation (Bissell and Hines, 2011; Bissell and Radisky, 2001). The TME refers to local environmental influences on tumor cells, including ECM composition (Iyengar et al., 2005; Provenzano et al., 2008), ECM mechanical stiffness (Levental et al., 2009; Paszek et al., 2005), and paracrine signaling with stromal cells (Grivennikov et al., 2010; Orimo and Weinberg, 2006). The TME provides continuous biochemical and mechanical feedback to resident cells within the vicinity of tumor cells, and evidence suggests that this complex milieu can promote or restrict metastatic progression (Fig. 2). With the TME metastatic model, disruptions to homeostatic balance between cancerous cells and the local environment accumulate to an extent that the environment itself serves as a driver of tumor proliferation and invasive behavior (Bissell and Hines, 2011; Bissell and Radisky, 2001).

In order to elucidate the mechanisms by which tumor cells acquire metastatic traits, researchers have made extensive use of *in vitro* and *in vivo* models. *In vitro* refers to models in which cells are cultured in an artificially constructed environment, whereas *in vivo* refers to models where cells exist in the native-like environment of an animal. The *in vitro* model can be sub-divided into 2D models and 3D models, where cells are cultured either on top of a flat support substrate such as standard tissue culture plastic or inside a 3D support substrate such as Matrigel (basement membrane-like ECM gel material). 2D *in vitro* models have served as the workhorse of biological discovery since the advent of tissue culture and continue to serve as a major tool for cell behavior investigations. Although the 2D model has led to many important discoveries, there is a growing recognition that 3D models allow recapitulation of aspects of tumor biology including cell proliferation, 3D cell migration, nutrient and waste diffusion kinetics, angiogenic recruitment, intravasation, and extravasation (Box 1) (Abbott, 2003; Griffith and Swartz, 2006; Yamada and Cukierman, 2007). 3D *in vitro* models are generally easier to image, more amenable to manipulation, less expensive and can be processed at a higher throughput compared with *in vivo* models (Burg et al., 2010). However, we must realize that *in vitro* models can only approximate the systems, cells and tissue of the body (Schuessler et al., 2014). *In vitro* models can, however, guide us to understanding underlying biochemical principles that can later be verified *in vivo*.

Primary tumors and their resident cells exhibit incredible heterogeneity (Box 1) in nearly every measurable characteristic, including cellular genotype, epigenetic state, matrix composition, metastatic potential and therapeutic resistance. Despite progress made with 3D *in vitro* models, heterogeneous TME models remain difficult to produce. The TME is not homogenous, and recent findings indicate that heterogeneity of environmental features is important for recapitulation of native tumor behavior. One key weakness of current 3D models is the lack of vasculature or complicated vascular structures to mimic blood vessel structures that are important for cancer cell interactions with endothelial cells during tumor proliferation, angiogenic recruitment and intravasation (Kolesky et al., 2014; Yamada and Cukierman, 2007). Blood vessels are also known to lead to the formation of oxygen diffusion gradients that can promote chemotactic tumor cell invasion (Mosadegh et al., 2015), promote angiogenic sprouting (Verbridge et al., 2013) and influence delivery of chemotherapeutics to solid tumors (Pàez-Ribes et al., 2009). For these reasons, vasculature should be incorporated into *in vitro* models to better recapitulate the native disease state (Miller, 2014).

Stromal cell recruitment is difficult to track with current models of metastasis. Boyden chamber assays utilize transwell chambers (standard tissue culture plate inserts) to co-cultivate two cell types (Boyden, 1962). Transwell chambers have been important for understanding migration of cancer cells, but such assays cannot recapitulate 3D clusters of tumor cells nor easily control the spatial distribution of cancer and stromal cells. Microfluidics methods to fabricate micro-scale fluidic channels have opened possibilities for improved *in vitro* models with controllable microenvironmental features (Young and Beebe, 2010; Zervantonakis et al., 2012) on the scale of 100 to 1000 µm thickness, which facilitates the use of fluorescent imaging methods for probing and measuring cancer cell behavior. However, traditional microfluidic fabrication techniques cannot easily fabricate 3D structures with increased thickness and thus cannot easily recapitulate the spatial heterogeneity of the ECM in 3D (Xia and Whitesides, 1998).

Three-dimensional printing (3DP) (Box 1) is one emerging method for fabricating 3D scaffolds that capture TME heterogeneity (Sears et al., 2016). 3D printing is a method for constructing physical 3D objects through additive manufacturing, in which material is deposited in discrete positions within a defined volume of interest – typically on the order of 0.5-10 ml. Whereas 2D printing is the patterning of flat patterns onto a starting surface, 3D printing can be described as the patterning of volumetric patterns into an empty space. During 3D printing, material is distributed into 2D patterns of material that stack together to form complex 3D shapes. Designed initially for the manufacturing of plastic prototypes and objects, researchers in the field of tissue engineering have been steadily adapting 3D printing methods for biological applications, leading to the emergence of ‘3D bioprinting’. Using similar physical principles, 2D patterns of biomaterials (Box 1) containing cells and other bioactive factors can be stacked to form 3D scaffolds that mimic native living tissue. 3DP excels in automation, precision, and reproducibility – key design goals in our search for techniques to pattern heterogeneous tumor models. The ability to pattern in 3D allows for fabrication of complex, heterogeneous tissue structures that recapitulate features of the microenvironment not possible through other tissue culture or microfluidics *in vitro* techniques.

In this Review, we discuss new biofabrication technologies based on 3DP and we suggest their potential utility in building *in vitro* models that can recapitulate TME heterogeneity. We first provide an introduction to TME heterogeneity and an overview of the fundamentals of 3DP. We next describe several 3DP methods currently used for tissue engineering applications that are relevant to fabricating *in vitro* tumor models with TME heterogeneity. For each technique, we discuss key considerations and limitations linked to these technologies and suggested applications for investigating metastasis. Finally, we discuss future directions of 3DP technology for tumor biology. We believe 3DP technologies will provide cancer biologists with a unique opportunity to investigate cellular physiology and disease progression *in vitro* with unprecedented control and reproducibility.

## Tumor microenvironment heterogeneity

The ECM, a key aspect of the cellular environment of a tumor, is a pervasive structural feature that surrounds all eukaryotic cells and serves an integral role in cell signaling and tissue organization (Hynes, 2009). Moreover, the ECM is constructed from a wide variety of molecular components; the exact composition is tissue-specific and significantly affects cell behavior (Mouw et al., 2014; Rozario and DeSimone, 2010). For embryogenesis and normal tissue homeostasis, ECM components direct cell differentiation (Gattazzo et al., 2014; Watt and Huck, 2013). In the context of tumor cell invasion, the ECM has been reported to inhibit (Bussard et al., 2010; Dolberg and Bissell, 1984; Weaver et al., 1997) or conversely, induce invasive behavior in cancer cells (Gill et al., 2012; Maffini et al., 2004; Shen et al., 2014) depending on ECM structural composition or matrix stiffness*.* We note here that, adding to this complexity, the ECM is not static. Interstitial cells are continuously degrading existing ECM and depositing new matrix molecules, and this continuous turnover varies depending on the tissue type and subcompartment (Wagenseil and Mecham, 2009). The ability to control *in vitro* tissue construct properties over time, termed ‘4D printing’, is outside the scope of this Review but the nascent field is growing rapidly (Sydney Gladman et al., 2016).

The TME interacts with cancer cells to influence metastatic progression based on environmental features (Fig. 2), including: (1) mechanical stimulation, governed by matrix stiffness, matrix porosity, local tension and compression on cells, and interstitial pressure (Giannelli et al., 1997; Levental et al., 2009; Polacheck et al., 2011; Yu et al., 2011); (2) cell–matrix interactions including integrin-mediated focal adhesion interactions, MMP-mediated matrix degradation, and matrix-tethered growth factors (Raeber et al., 2005; Reynolds et al., 2009; Yu and Stamenkovic, 2000); (3) cell–cell interactions with surrounding stromal fibroblasts and pro-inflammatory immune cells (Grivennikov et al., 2010; Wyckoff et al., 2004); (4) oxygen, nutrient, and soluble cytokine gradients (Eccles, 2005; Kim et al., 2010; Strieter et al., 2004); and (5) tissue architectural features such as blood vessels, angiogenic sprouts and endothelial barriers (McDonald and Baluk, 2002; Papetti and Herman, 2002).

Intercellular communication coordinates a variety of cancer hallmarks including invasion, and tumor-promoted inflammation (Hanahan and Weinberg, 2011). Tumor invasion refers to the pathophysiologic migration of tumor cells into surrounding tissue. Invasion can be partially attributed to cross-talk signaling between tumor cells and macrophages (Condeelis and Pollard, 2006; Yamaguchi et al., 2005) or fibroblasts (Kalluri and Zeisberg, 2006; Karagiannis et al., 2012). Cross-talk signaling can lead to secretion of matrix metalloproteinases (MMPs) that degrade local ECM to clear pathways for tumor cell invasion, can cause inappropriate activation of epithelial-mesenchymal transition programs, and can cause chemotactic migration towards blood vessels (Hanahan and Coussens, 2012). Chronic inflammation from infections such as hepatitis is associated with tumor sites, and inflammatory cells are often found at primary tumor sites (Balkwill and Mantovani, 2012; Grivennikov et al., 2010; Landskron et al., 2014). Some inflammatory cytokines directly promote invasion and metastasis (Coussens and Werb, 2002). Paradoxically, in some cases macrophages and other immune cells can inhibit tumor progression (Coussens et al., 2013). Better *in vitro* systems could help uncover the multiple roles of stromal cell interactions with cancer cells and thus provide insight into the mechanisms underlying these cancer hallmarks.

Metastatic dissemination of cancer cells to distant sites occurs primarily via blood vessel networks (Chaffer and Weinberg, 2011; Chambers et al., 2002). Initially, angiogenesis forms new blood vessels that add nutrient supply lines to improve tumor growth rate. These incompletely formed vessels can serve as ‘leaky’ entrances for tumor cells to invade the bloodstream (Chambers et al., 2002; Roskoski, 2007). Under physiologic conditions the ‘angiogenic switch’, or balance between contributions of pro- and anti-angiogenic signals, remains ‘off’ unless external agents like tumor cells force the balance in favor of angiogenesis (Carmeliet and Jain, 2000; Folkman, 2002). Tumor cells accomplish this in part by paracrine signaling with endothelial cells to secrete vascular endothelial growth factor (VEGF) and other pro-angiogenic factors (Dankbar, 2000). After intravasation into the bloodstream, circulating tumor cells somehow infiltrate other types of tissue to establish a secondary colony. Secondary organ site locations for metastatic lesions are non-random for some types of primary tumors (Paget, 1989), and tissue infiltration by circulating tumor cells is highly inefficient (Cameron et al., 2000; Luzzi et al., 1998), both of which indicate an opportunity for studying secondary site environmental features that promote or inhibit tumor proliferation. Thus, hollow blood-vessel-like structures are key to studying metastatic dissemination *in vitro*.

In light of the evidence implicating the environment surrounding a tumor as contextually promoting or inhibiting tumor behavior, the development of *in vitro* models with controlled heterogeneity will be pivotal to further elucidate the etiology of metastatic disease. There are several key features an *in vitro* model needs to better mimic the native TME. Fundamentally, any *in vitro* model for metastasis should be three-dimensional because of the dynamics of diffusion (cytokines, nutrients, waste) and migration (tumor invasion, inflammatory cell recruitment). Such a 3D model should be composed of an ECM-mimetic material with tunable mechanical and bioactive properties to recapitulate cell-ECM interactions. Paracrine communication between tumor and stromal cells influences angiogenesis, migration and inflammatory cell recruitment as discussed above, which means that the ideal *in vitro* model should enable two or more cell types to be included. Blood vessels and lymphatics are crucial to intravasation and extravasation, so a perfusable tube or branching network would further improve an *in vitro* model for metastasis.

## An overview of 3D bioprinting

3DP has emerged as a revolutionary technique for rapidly prototyping new designs for products useful to a myriad of fields. The origins of 3DP can be traced to a patent application from 1984 by Charles W. Hull (Hull, 1986), which describes a system for building 3D objects from repeated patterning and stacking of 2D cross-sections of a photopolymerizable fluid. Since the 1980s, the idea of 3DP has been expanded by developing new machines capable of printing by different methodologies with a broader range of materials. Applications for 3DP now span an incredibly wide range of fields including the arts, commercial product design, large-scale industrial manufacturing and construction, and more recently biomedical and biological applications.

**Fig. 3.**
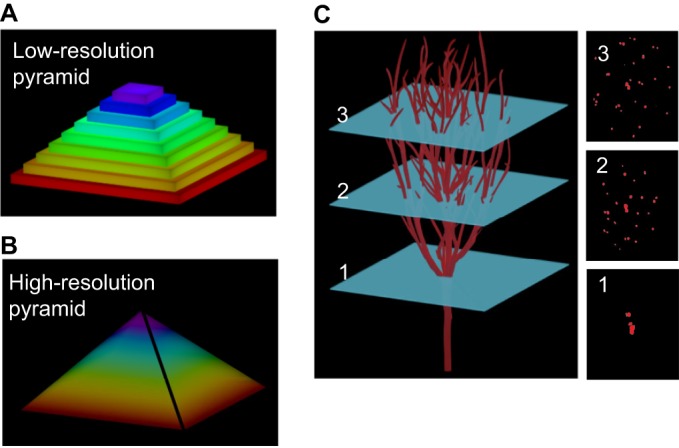
**Layer-by-layer 3D printing.** A common strategy for constructing three-dimensional objects is layer-by-layer construction, whereby a 3D structure is formed by stacking several layers of flat materials into a 3D pattern. Each layer can be thought of as a 2D pattern that has been expanded slightly into a thin 3D volume. An easy, illustrative example is provided by the formation of a pyramid shape. Each layer in a pyramid is a square 2D pattern with limited volume. (A) A low-resolution 3D object refers to an object formed from thick layers, which for a pyramid results in an object with thick, prominent steps. (B) By increasing the number of layers and decreasing thickness, the resolution of the pyramid is increased to give the appearance of a smooth surface. (C) For 3D bioprinting, complex structures such as vasculature can be constructed layer-by-layer with feature resolution dependent on layer thickness. Left panel shows an example 3D object representing a branching vascular structure is depicted. The vascular object can be constructed through iterative addition of 2D patterns. Right panel examples 1, 2 and 3 show top-down views of select 2D patterns at differing layers heights in the object.

3DP refers to a subset of techniques from the more general category of additive manufacturing, a process by which objects are formed by additively joining material into a 3D pattern (Miller, 2014). Typically, ‘2D’ cross-sections (3D volumes with relatively small thickness dimension) are incrementally stacked on top of one another to form a 3D patterned structure (Fig. 3). Other methods of printing that do not rely on 2D stacking of materials exist (Hinton et al., 2015; Wu et al., 2011), but these methods are not discussed in this Review. 2D patterns can be positioned by hand; however, manual alignment and stacking of successive layers quickly becomes a critical impediment (Gurkan et al., 2013). The commoditization of electronic and robotic equipment has facilitated the design of dozens of types of additive manufacturing that benefit from high precision and automation not typically available in a research lab. Common methods for positioning the addition of new material can be droplet addition over 2D arrays such as by an inkjet printer (Gurkan et al., 2014; Li et al., 2015), extrusion (Box 1) through a nozzle along linear paths (Pati et al., 2014; Zein et al., 2002), polymerization by 2D laser rastering (Hribar et al., 2014; Neiman et al., 2015), and light projection in 2D patterns (Elomaa et al., 2015; Melchels et al., 2010). New material is solidified or adhered to the previous layer by one of several general methods including thermal phase transitions, chemical cross-linking reactions and light-based polymerization reactions. The complicated nature of material physical properties, adhesion mechanisms and patterning techniques renders optimization of relevant parameters necessary (Knowlton et al., 2015; Tasoglu and Demirci, 2013).

3D bioprinting simply refers to the application of 3DP to a biological application. 3D bioprinting applications from the past decade have included engineering implantable tissue scaffolds (Sooppan et al., 2016) as well as *in vitro* tissue scaffolds for studying stem cells, co-culture tissue models and tumor microenvironments (Gurkan et al., 2014; Kang et al., 2016; Pati et al., 2014). For all bioprinting applications, the goal is to control the patterning of both cells and biomaterials into tissue-like structures. Biocompatibility is the most important factor to consider in 3D bioprinting design, which means that materials, methods to add materials, and material adhesion mechanisms (such as thermal cooling and cross-linking) must all be non-toxic and non-destructive to cells. 3DP was designed for hard, dry plastic manufacturing rather than soft, wet biological tissue, providing design constraints that necessitated re-engineering of 3DP techniques from the ground up. Commercial printers with standardized biological printing materials do exist, but many biological applications also make use of 3D printers and accompanying software that are custom designed. Here, we describe some of the more notable developments in 3D bioprinting. We also note that many groups have developed *in vitro* cancer models that are manually assembled, and are therefore ripe for translation to a more reproducible additive biomanufacturing platform (Bray et al., 2015; Kaemmerer et al., 2014; Loessner et al., 2013; Loessner et al., 2016; Riching et al., 2015).

## 3D printing of heterogeneous microenvironments

### Biomaterial considerations

The choice of biomaterial is one of the first considerations for developing an *in vitro* model that mimics the native ECM. The ECM is constructed from complex combinations of several classes of proteins and other molecules (Rozario and DeSimone, 2010) and consequently, ECM mimetic constructs with identical biochemical and structural properties are difficult to produce. Cell compatibility with the biomaterials and polymerization mechanisms also impacts on the choice of biomaterial, and compatibility with a 3DP method adds further constraints to the types of biomaterials that can be used. Nonetheless, a variety of biomaterials have been developed that can be used to fabricate 3D *in vitro* scaffolds by 3DP. These materials can be divided into natural, synthetic or hybrid natural/synthetic materials (Hutmacher, 2010; Sionkowska, 2011).

‘Natural materials’ refers to a category of biomaterials that are derived from living sources. Matrigel^®^, an ECM-based material isolated from Engelbreth-Holm-Swarm (EHS) tumors in mice, is one of the most commonly used natural biomaterials (Kleinman and Martin, 2005) and has been particularly useful for *in vitro* studies on invasive behavior of tumor cells (Petersen et al., 1992; Weaver et al., 1997). Additionally, collagen I, gelatin, hyaluronic acid (HA), fibrin, alginate and chitosan can also serve to build 3D scaffolds (Murphy and Atala, 2014; Tibbitt and Anseth, 2009). Natural biomaterials (especially Matrigel) generally reflect the native *in vivo* cellular ECM composition better than synthetic materials owing to the pre-existing complexity of sources for natural materials (Kleinman and Martin, 2005).

Synthetic biomaterials are artificial materials such as poly(ethylene glycol) (PEG), poly(n-isopropylacrylamide) (pNIPAAm), and poly(caprolactone) (PCL) that are suitable scaffold materials for 3D cell culture (Gill and West, 2014). With little or no inherent bioactivity, these biomaterials can be extensively modified to selectively add bioactive components to mimic natural ECM properties (Zhu, 2010). Short peptide sequences like the commonly used arginine–glycine–aspartate (RGD) motif can be immobilized to synthetic hydrogels to present integrin binding sites that promote cell adhesion and cell proliferation (Hersel et al., 2003; Ruoslahti, 1996). Selective ECM degradation by MMPs can be achieved by incorporating MMP-cleavable peptide sequences into the hydrogel backbone (Raeber et al., 2005). Other basic growth factors like transforming growth factor beta 1 (TGFβ1), TGFβ2, and basic fibroblast growth factor (bFGF), can be immobilized to hydrogel scaffolds to alter the behavior of encapsulated cells (Bentz et al., 1998; DeLong et al., 2005; Mann et al., 2001).

ECM mechanical properties such as matrix stiffness can be controlled through biomaterial choice and functionalization. Biological tissues vary widely in stiffness, ranging from soft tissue in the brain (∼0.1 kPa) to very stiff tissues in bone (∼80 kPa) (Guvendiren and Burdick, 2013). In the past decade, research has revealed that matrix mechanical properties can drastically change cell behavior including stem cell differentiation (Engler et al., 2006) and tumor migration (Chaudhuri et al., 2015; Zaman et al., 2006). The stiffness of synthetic or modified natural materials can be tuned by controlling polymerization reaction conditions (DeForest et al., 2010; Gill et al., 2012).

### Material-extrusion-based 3D bioprinting

Aspects of TME heterogeneity can be recapitulated with 3D­-printed *in vitro* models using extrusion and inkjet bioprinting (Box 1). For extrusion and inkjet 3D printing, bioinks (Box 1) composed of biomaterials, cells and soluble factors are selectively patterned onto a surface to form 3D scaffolds. By changing the composition of the bioink, cell type and soluble factors can be readily exchanged to produce *in vitro* scaffolds with a heterogeneous composition. Printing with a single bioink can generate structures with architectural features such as hollow channels. Expansion to two or more bioinks allows users to spatially pattern ECM materials and cells, enabling the creation of *in vitro* models with heterogeneity that is not easily achieved using scaffolds formed from a single homogenous mixture.

**Fig. 4.**
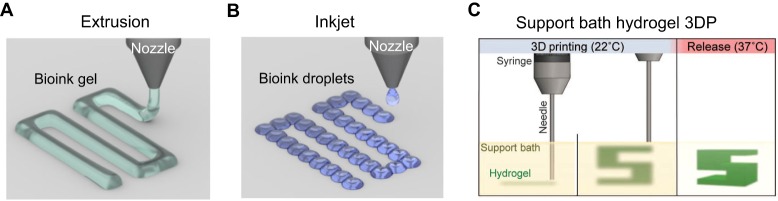
**Material extrusion-based 3D bioprinting.** (A) For extrusion-based bioprinting, material is selectively guided onto a platform via pressurized emission through a nozzle. The material, or ‘bioink’, is composed of an ECM-like biomaterial, cells and soluble factors. (B) For inkjet-based bioprinting, droplets of bioink are distributed across a surface to form a patterned layer. (C) For support bath hydrogel 3DP, biomaterial is extruded into a support hydrogel material. At 22°C, the hydrogel bath is stable enough to support the extruded print material, but at 37°C, the hydrogel bath transitions into a more liquid state to release the 3D printed object. The support bath allows formation of complex structures with overhanging regions such as the 3D ‘S’ structure, which is not possible with regular extrusion 3DP. Additionally, support bath hydrogel 3DP enables fabrication of structures without the need for layer-by-layer production; material can be extruded along any linear path within the enclosed gel bath volume. Reproduced with permission from Hinton et al. (2015).

In typical extrusion-based 3D printing, small amounts of bioink are deposited onto a platform by forcing material through a nozzle in a controlled, continuous stream (Pati et al., 2015). The material-dispensing system can freely move in the *x*- and *y*-directions to deposit material in 2D patterns onto a support platform (Fig. 4A). This platform can additionally move in the *z*-direction to allow sequential addition of 2D patterns, which stack to form a 3D scaffold. Recently, Shim et al. (2012) built a multimaterial extrusion 3D printer, called the multi-head tissue/organ-building system (MtoBS), which employs six nozzles capable of incorporating up to six bioinks into a single 3D scaffold. The bioprinter functions by alternating between support layer ‘walls’ of a stiff material, PCL, with layers of a softer alginate gel that is less structurally stable but capable of supporting encapsulated cells. Later work adapted the MtoBS to additionally print with soft, decellularized matrix materials capable of promoting human mesenchymal stem cell (hMSC) differentiation (Pati et al., 2014). Extrusion-based 3DP has been applied for the fabrication of vessel-like constructs. One such example makes use of calcium-mediated polymerization of alginate to directly form hollow, vessel-like structures (Grolman et al., 2015). With this specialized printer, a central calcium chloride stream is co-extruded with a surrounding alginate solution, which leads to polymerization in a hollow cylindrical structure at the solution interface.

Inkjet bioprinting is a related 3DP method in which tiny volumes of bioink in the form of droplets are sprayed onto a surface, much like 2D inkjet printing (Fig. 4B) (Derby, 2008). Li et al. (2015) recently reported an inkjet-based method of printing cell-laden hydrogels using peptide-DNA and DNA cross-linker cell suspensions via nanoliter droplets to form multi-layer hydrogels. Although the authors did not demonstrate printing with more than two nozzles, the addition of one or more nozzles could allow patterning of multiple cell types. Gurkan et al. (2014) demonstrated a similar printing technique that can be used to form objects from droplets of bioinks composed of the photopolymerizable GelMA, hMSCs and either transforming growth factor beta 1 (TGF-β1) or bone morphogenetic protein 2 (BMP-2). TGF-β1 and BMP-2 have both been previously reported to promote osteogenic and chondrogenic differentiation in hMSCs (Dickhut et al., 2010; Pittenger, 1999). When these two bioinks were printed in an interlocking pattern to form a spatial gradient, expression markers for both chondrogenic and osteogenic differentiation were significantly upregulated compared with single growth factor controls (Gurkan et al., 2014). A key goal in cancer research is to identify specific matrix factors such as chemical ligands and mechanical stiffness that might impinge on or correlate with metastatic progression (Liu et al., 2012; Yu et al., 2011). Bioprinted tumor models might help uncover new therapeutic targets to inhibit or antagonize these specific interactions.

Most 3DP techniques are unable to print truly ‘freeform’ objects, where there are no spatial restrictions on the shape of the object. These limitations stem from the inability to deposit material at a point that is not directly connected to a previous section of the object. An example would be attempting to print the shape of a palm tree by starting with the base of the tree – the tips of the hanging branches would be impossible to start in mid-air. A solution to this problem is to utilize a support material that can physically support printed material at any volumetric point. Extrusion printing inside a support bath of hydrogel material has emerged as a solution to freeform printing. The key is the use of material combinations that permit extrusion of material but prevent material displacement post-extrusion.

Recently, true freeform structures have been formed by extrusion bioprinting into a support material using a technique called hydrogel support bath 3DP (Fig. 4C). One major advantage of hydrogel support bath 3DP is the ability to generate hollow networks of tubes that resemble vasculature. Hinton et al. (2015) directly extruded material into a gelatin microparticle bath to form 3D structures. The gelatin presents low resistance to shear stress (i.e. extrusion nozzle moving) but high resistance to normal forces (i.e. supporting extruded material against gravity) (Hinton et al., 2015). Using alginate, the authors demonstrate printing of an elastic miniature of the human femur, and a hollow branching network. Bhattacharjee et al. (2015) used a similar method with a soft granular gel support bath that is natively rigid but able to fluidize with high shear stress. This property combination allows material to be easily deposited by extrusion, but will cement previously extruded material rigidly in place. The extruded gel can be photopolymerized into a stable continuous structure. As an extrusion-based technique, support bath 3DP can also be used to generate cellular and soluble factor heterogeneity. Multiple nozzles or a complicated multi-reservoir system would allow multiple materials to be patterned in 3D.

Extrusion and inkjet bioprinting share many related design considerations and limitations for 3DP. Often, ECM and cellular heterogeneity can be simultaneously achieved because existing bioprinting applications have been optimized for printing material with encapsulated cells. Physical considerations for these printing methods are complicated and have been reviewed previously (Knowlton et al., 2015; Murphy and Atala, 2014). Key limitations for novel tumor engineering applications will be optimizing fluid mechanics for material extrusion and phase transition of the material post-extrusion. For techniques with nozzle extrusion, hydrodynamic forces on the cells resulting from nozzle width and roughness, cell size, and cell medium composition and flow properties need to be considered. Viscoelastic properties will vary among biomaterials, which fundamentally changes the flow rate of the material in response to the extrusion or ejection method. Furthermore, the polymerization mechanism changes the timing of material extrusion, as well as fundamental aspects of the printing apparatus such as temperature control for thermo-phase transitions or properties of light for photopolymerization. One major benefit for tumor modeling applications is the resilience of cancer cells to mechanical stressors during ejection or gel encapsulation compared with non-cancerous cells. Similar arguments can be made for inkjet droplet bioprinting with additional considerations for droplet temperature during ejection and mechanical forces of droplet impact (Knowlton et al., 2015).

An additional consideration for multimaterial extrusion and inkjet printing is the number of distinct materials, which is limited by the number of nozzles or inkjet cartridges. Traditional color inkjet printers have four or more ink cartridges, which facilitates the development of printing heterogeneous materials, but the thermodynamic restrictions of droplet formation limit printable materials. Nozzle extrusion printers have more flexibility with material deposition; however, multiple material streams are more difficult to design and build. Moreover, deposition of one material could be incompatible with other potential co-printed materials. Natural biomaterials that undergo a reversible phase transition from gel to solid are ideal biomaterials for extrusion 3DP, whereas liquid biomaterials that can be chemically cross-linked are better suited for inkjet 3DP. Owing to constraints on biomaterials, the printing resolution of features is on the scale of 200 µm (Miller, 2014).

### Light-based 3D bioprinting

Light-based 3DP methods are another major technique for fabricating 3D scaffolds. Broadly, stereolithography (SLA) (Box 1) encompasses techniques that utilize light in the form of a focused laser or a 2D projection to initiate a light-based polymerization reaction. The transition from liquid to solid is limited to regions where the material has been exposed to light of a specific wavelength. Several synthetic biomaterials can undergo light-based polymerization reactions that do not prohibitively affect cell viability, which allows cells to be encapsulated in the bulk material. The use of synthetic biomaterials additionally allows bioactivity and scaffold mechanical properties to be readily controlled. Additionally, scaffolds with hollow channels are easy to produce via light-based 3DP, which can be perfused with a nutrient source to support higher densities of cells throughout the scaffold.

**Fig. 5.**
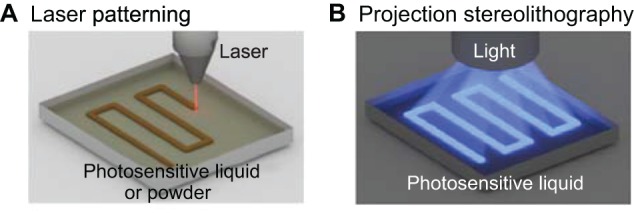
**Light-based 3D bioprinting.** (A) In laser patterning, a laser is focused onto singular points to locally photopolymerize material. The laser beam can be rastered across the surface to create 2D patterns of material. In a similar technique, selective laser sintering (SLS, not shown), a laser is used to fuse powder material together to form 2D patterns of material. SLS is particularly important because each layer is fully supported by the sintered or un-sintered powder of the previous layers, which permits freeform 3D printing of structures. (B) With projection stereolithography, a 2D pattern of light is directly projected onto a photopolymerizable material to form entire layers in singular steps. Projection stereolithography is notable in that each layer is formed with constant time, regardless of pattern complexity or shape.

With laser-based 3DP, patterns of material are traced by a laser capable of planar motion. In one technique termed laser stereolithography, the laser can either directly cure patterns into a photosensitive medium, and an independent *z*-axis stage can then be moved to pattern successive 2D layers of materials to form a 3D shape (Fig. 5A) (Hribar et al., 2014). In one application of laser stereolithography, PEG diacrylate (PEGDA) was photopolymerized by a UV laser to form small arrays of channels for cultivating hepatocytes. Using laser scanning, the hydrogel was polymerized into rectangular or ellipsoidal channel shapes, and size, aspect ratio, positioning and depth could be controlled (Neiman et al., 2015). Another technique is called laser-induced forward transfer of liquids or LIFT, which describes a technique for using a laser to force small droplets of biomaterial from a substrate onto a separate platform or object (Colina et al., 2006; Gruene et al., 2011). This technique operates similarly to inkjet bioprinting, with a focused laser rather than a nozzle used to form droplets. Guillotin et al. (2010) demonstrated the usefulness of LIFT by printing with a high cell density alginate bioink. ‘Ribbons’ coated with bioinks of various compositions could be interchanged to fabricate concentric cylinders of multiple distinct cell types. The laser allows for rapid ejection of biomaterial droplets, which provides a distinct advantage; however, the complexity and fidelity of the resulting 3D scaffolds is limited by difficulties in reliably controlling droplet deposition.

Digital light processing (DLP) stereolithography refers to the use of 2D projections of light to pattern layers of a 3D scaffold. With a distributed light source, whole 2D patterns are simultaneously projected onto a photopolymerizable material (Fig. 5B). An independent *z*-axis stage can be moved to iteratively polymerize layers of hydrogel to form a 3D scaffold (Melchels et al., 2010). In one example, light can be blocked by a physical sheet with a stenciled pattern, called a photomask, to form a pattern of light. Gurkan et al. (2013) described a heterogeneous hydrogel formed via successive photomask steps with different hydrogel materials to construct heterogeneous layers, and *z*-axis motion can augment this technique to produce 3D scaffolds with depth. The resolution of the printer allows users to mass-produce up to 100,000 3D scaffolds during a single round of printing. However, a major drawback to this technique is the complications associated with layer alignment (LaFratta et al., 2006), which requires photomasks to be aligned with micro-scale precision. An alternative to blocking light with a photomask is to use a common video projector to illuminate patterns onto a photosensitive material. Elomaa et al. (2015) built a DLP-stereolithography 3D printer that projects light down into a reservoir of a biocompatible hydrogel material. The authors were able to print a toroid shape with encapsulated human umbilical vein endothelial cells (HUVECs) as well as a large, bifurcating vessel junction.

Albrecht et al*.* (2006) demonstrated an early method of patterning cell types in 3D by dielectrophoretic cell patterning (DCP). With this technique, cells arrange into patterns according to dielectrophoretic forces generated by alternating currents across a cell suspension. Essentially, the electrical current causes the cells to move, akin to gel electrophoresis. After patterning, cell positions are locked by photopolymerization of the pre-polymer material. Multiple cell types can be patterned into a 3D structure by repeated DCP application steps where multiple layers of hydrogel are successively formed. The authors applied the approach to show that microscale organization of chondrocytes influences ECM secretions, whereas randomly distributed chondrocytes have no effect. This technique provides a powerful method for patterning tumor and stromal cells into microscale 3D patterns with layer-by-layer (Box 1) iterative DCP fabrication. A major drawback to this method is that the layers are subject to non-uniform illumination, which affects the duration of polymerization and thereby gives rise to non-uniform mechanical stiffness throughout the layers. Additionally, this process restricts heterogeneity of cell type, soluble factors and ECM composition, as only one condition can be applied for each layer along the *z*-axis.

Multiphoton excitation (MPE) is an imaging technology that has been adapted to pattern sub-micron scale features into *in vitro* 3D constructs (Xing et al., 2015). MPE refers to an infrequent event during which two or more photons simultaneously excite the same molecule, resulting in a lower effective wavelength than the original source wavelength. During MPE imaging, high-energy laser pulses are focused into a small focal region that contains a high density of photons. In this region, the frequency of MPE events can excite a sufficient number of fluorescent molecules to be detected by microscopy (Li and Fourkas, 2007). Miller et al*.* (2006) demonstrated an early application of MPE imaging, which uses an MPE microscope to initiate a light-based polymerization reaction within the laser focal region. Ovsianikov et al. (2010) presented another interesting application of multiphoton excitation to fabricate hydrogel scaffolds containing heterogeneous cell distributions. The scaffold is first formed in a reservoir of photocurable material, then the scaffold is seeded using LIFT.

Recent advances in multiphoton imaging technology and biochemistry have also enabled post-printing modifications to a 3D scaffold. Molecules have been developed that can covalently bond a hydrogel at one excitation wavelength and later be cleaved by another excitation wavelength. This allows MPE-based spatiotemporal addition or removal of materials in 3D scaffolds, referred to as a ‘4D’ model (DeForest and Anseth, 2011, 2012; Luo and Shoichet, 2004). A similar light-cleavage reaction was employed by Mosiewicz et al. (2014) to achieve matrix stiffness patterning in 3D.

Photolithographic methods for 3DP are distinguished by the use of photopolymerization to add new layers to an object, which offers its own strengths and limitations. Like extrusion and inkjet printing, photolithography permits co-printing of multiple biomaterials and multiple cell types. One major strength of printing with light is the ability to specify 2D patterns of material addition or rapidly raster a focused laser beam, which can significantly decrease the duration of printing compared with techniques relying on the physical extrusion of material. However, the time required for material addition to the platform and subsequent polymerization can lead to cell sedimentation. Neutral buoyancy solutions can correct for cell sedimentation, but formulating such solutions can be difficult and might restrict biomaterial choices. The requirement of light-initiated polymerization limits the biomaterial choices to synthetic biomaterials. Furthermore, reaction conditions must be compatible with cell biology, which restricts reaction conditions including light wavelength and exposure time as well as photoinitiator toxicity. Despite the light­exposure constraints, the range of exposure times enables fabrication of scaffolds with heterogeneous mechanical stiffness because increased exposure time will increase gel stiffness. Moreover, cancer cells might be more tolerant of phototoxicity than primary cells, mitigating complications from light exposure in the generation of bioprinted tumor models.

One key benefit and limitation to multiphoton microscopy is the size scale for patterning. Multiphoton microscopy can only modify small voxels (volumetric units) on the order of 1 µm^3^ (Li and Fourkas, 2007), which both permits microscale feature patterning and restricts the effective patterning to microscale features in small (mm) gels. Another key limitation to multiphoton patterning is the limited availability of light-based chemistries that are orthogonal, compatible with cells, and adaptable to a wide range of molecules (DeForest and Anseth, 2011).

### Sacrificial template 3D bioprinting

**Fig. 6.**
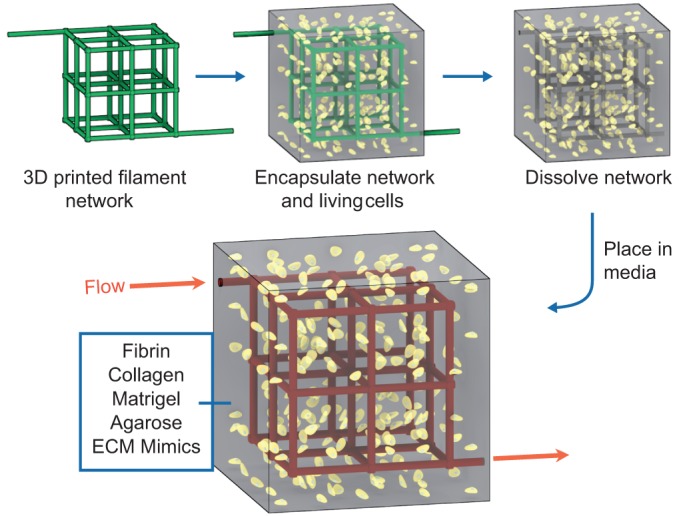
**Sacrificial template 3D bioprinting.** An alternative method to ‘positive-space’ 3D printing is sacrificial template 3DP. For this method, a template material is formed into a 3D scaffold by a standard 3DP method. The product scaffold is cast with a biomaterial containing cells and/or soluble factors, and then the template material is removed by chemical dissolution or physical dislocation. In this example, a carbohydrate glass lattice (green) is fabricated via extrusion-based 3DP then encapsulated in ECM (gray) containing live cells (yellow). After the ECM solidifies, the sacrificial lattice is then dissolved, and the revealed vasculature can be perfused with media (red) to keep encapsulated cells alive. Reproduced with permission from Miller et al. (2012).

The 3DP methods presented thus far have all been examples of ‘positive-space printing’, where the final 3D object is directly formed during the printing procedure. In contrast, ‘negative-space printing’ or ‘sacrificial template 3DP’ (Box 1) generates final objects by first casting material around a 3D printed object, then dissolving or physically removing the 3D printed ‘negative’ object (Fig. 6). In other words, the goal is to print an object that corresponds to regions of empty space in the final desired 3D object. The key to this method of object fabrication is the material choice. The printing material must maintain a defined shape during the casting process and be selectively removable after casting is complete. Sacrificial template 3DP is particularly advantageous for generating hollow networks to mimic native vasculature. With positive-space printing, there can be difficulties with printing hollow, circular tubes because of issues with properly supporting overhangs at the points where the tube reconnects (i.e. like building an arched doorway). Moreover, the amount of time required to print a sacrificial template can be much shorter compared with the time required to print the surrounding volume.

One strategy for making blood vessels via sacrificial template 3DP is demonstrated by Bertassoni et al. (2014) who used extruded agarose cylinders to form a template for hydrogel casting with gelatin methacrylate (GelMA). Agarose does not adhere to polymerized gelatin methacrylate, which permits easy agarose extraction by vacuum aspiration. Such a technique can fabricate some degree of three-dimensionality, including limited blood vessel branching, but vasculature with multiple branching nodes are not feasible to produce with this method. Kolesky et al. (2014) also demonstrated an extrusion bioprinter capable of spatially patterning multiple cell-laden bioinks, including GelMA and Pluronic F-127, that can be sacrificed via temperature-dependent phase transition from gel to liquid. Additionally, Miller et al. (2012) used extrusion bioprinting to fabricate templates made of a carbohydrate glass, which are used to cast hydrogels. The carbohydrate glass composite can be dissolved with any water-based material including cell media. Carbohydrate glass can be printed with features like vessel junctions, but structures are limited to lattice-like architectures. Even with simple 3D vessel structures, sacrificial template printing has been shown to improve differentiation (Bertassoni et al., 2014) as well as improve angiogenic sprouting and the survival of fragile hepatocytes (Miller et al., 2012).

Another method of sacrificial template fabrication makes use of laser sintering (Box 1) to form the sacrificial scaffold. During selective laser sintering (SLS), neighboring granules of a powder material can be fused using heat generated by a focused laser (Fig. 5A) (Shirazi et al., 2015). For 3DP applications, 2D patterns can be sintered into powder, then a new powder layer can be added by lowering the previous layer and adding a fresh layer of powder over the existing object. Objects can be built layer-by-layer by ensuring that the successive layers fuse to the previous layer. Kinstlinger et al. (2016) recently used SLS to sinter PCL into 3D objects that were subsequently cast in PDMS. The PCL could be sacrificed using an organic solvent, leaving behind a hollow structure with potential use as a vasculature mimic. Although the use of the organic solvent is undesirable because it limits choice of materials for encapsulation, SLS printing utilizes a support structure that enables fabrication of 3D objects that cannot be made using traditional extrusion-based printing methods.

Template casting and hydrogel support bath 3DP are excellent techniques for building 3D *in vitro* hollow vessel structures, but there are limitations. The hollow space can be perfused, which improves nutrient availability and waste removal for supporting higher density cell populations. However, the bulk hydrogel cast around the sacrificial material will be uniform in ECM material and cellular composition and thus cannot recapitulate spatially heterogeneous native tissue. Existing techniques are limited in number and can only utilize a few biomaterials with special properties. Moreover, current 3DP capabilities can produce vessel diameters on the order of 100 µm and thus cannot achieve capillary level resolution of less than 10 µm.

## Outlook: challenges and opportunities

As outlined in this Review, recent research has clearly demonstrated the remarkable power of 3D bioprinting to improve fabrication of *in vitro* models. In keeping with its original purpose of rapidly prototyping new 3D objects, the adaptation of 3D printing for bioprinting applications has enabled biologists to rapidly prototype custom-designed 3D scaffolds for cultivating cells in a heterogeneous microenvironment (Table 1).

**Table 1.**
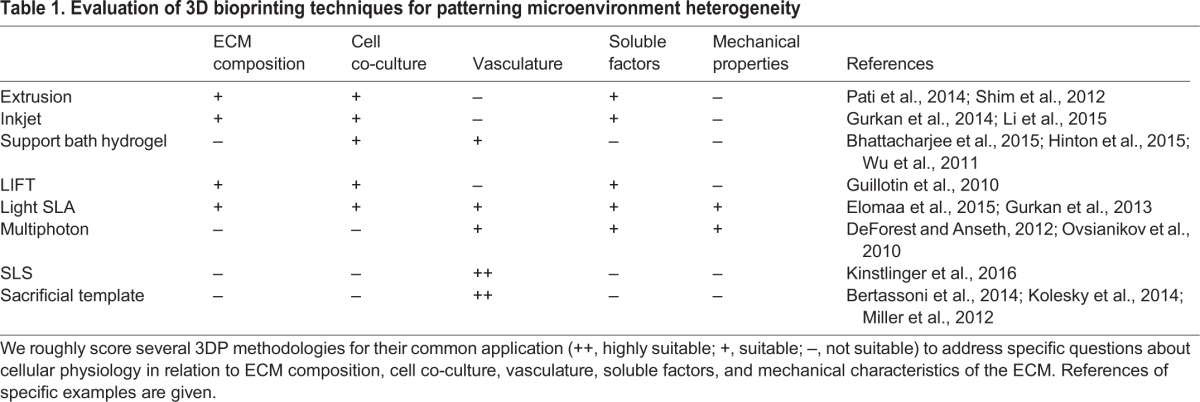
**Evaluation of 3D bioprinting techniques for patterning microenvironment heterogeneity**

With increasing recognition of TME heterogeneity as a major player in metastasis, further adoption of technologies including 3D bioprinting will be crucial to advance the field. A recent strategic workshop for developing improved systems for cancer research has summarized many aspects of the TME that are key to advancing *in vitro* modeling of cancer (Schuessler et al., 2014). For example, research is being conducted across multiple length scales [e.g. intracellular molecular interactions (nm), intercellular communication (µm), macro-tumor tissue architecture (mm-cm)] and multiple time scales [e.g. enzyme kinetics (ns-µs), changes in protein expression (min-h), metastatic progression (days-years)]. Further, the role of ECM mechanical and chemical composition as well as cross-talk between cancer cells and nearby stromal cells are providing new perspectives on disease progression and therapeutic targets (Schuessler et al., 2014). 3D bioprinting can address all of these issues, to varying degrees. Light-based, hydrogel support bath, and sacrificial template 3DP methods have all been employed to create 3D scaffolds with hollow, perfusable networks that can serve as blood vessel mimics. Light-based printing techniques can also pattern gradients of mechanical stiffness, which can be used to examine mechanical contributions of the ECM on local invasion by cancer cells. Extrusion, inkjet and stereolithography 3DP can construct 3D scaffolds with micro-scale resolution, and multiphoton emission techniques extend this range to nano-scale feature patterning. Advances in multimaterial 3D printing have further enhanced our ability to replicate the TME through patterning of multiple bioinks composed of ECM-like biomaterials, soluble signaling factors and cells. These bioinks can be used to form gradients of soluble or tethered bioactive molecules; cell co-culture models with controlled spatial arrangement; and scaffolds with complex ECM composition.

In the future, we can expect to see more examples of 3D bioprinting application to fabricate *in vitro* models of metastasis. A challenge in systems engineering is the tendency toward ‘over-engineering’ – adding more complexity than necessary – which can rapidly lead to an unwieldy or difficult-to-use workflow. However, it is clear that many current systems are too simple. We must be discrete in the exact characteristics we would like to model in an *in vitro* setting, and these specifics can also help dictate or recommend 3D bioprinting methodologies that can help us to achieve the desired tissue construct. By defining the simplest 3D model system for a specific study, the key environmental causes or modulators of cancer cells will be easily uncovered through standard hypothesis-driven research. 3D bioprinting could be used to achieve this goal given the potential for rapid prototyping and control over scaffold bioactive-signaling properties. Each of the variables can be manipulated and tested with high turnaround time to establish individual or combination influences on cancer behavior. 3D bioprinting enables reproducible fabrication of complex *in vitro* models with medium to high throughput, which improves our ability to reliably screen for aspects of the TME that contribute to the development of metastatic disease. In the context of metastatic disease, cancer cells are known to clearly change behavior over time, exhibiting invasion into the bloodstream or lymphatics and colonization (Box 1) and proliferation at secondary tumor sites. 3D printed models enable 4D manipulation of variables, which is crucial because cancer is a disease that unfolds over time and space. 3DP models allow control over 4D models such as patterned mechanical stiffening or softening, timed and localized release of growth factors from the surrounding matrix, and controlled perfusion profiles into vasculature.

There are still limitations to widespread adoption of 3D bioprinting by non-specialist cancer biologists for investigating metastasis. One of the main difficulties for *in vitro* models in general is the difficulty with tying *in vitro* cell behavior to *in vivo* cell behavior. However, this is a major problem with all *in vitro* testing methods, and 3D bioprinting does offer the ability for rapid turnaround testing of multiple scaffold types at a throughput that can provide definitive answers. Biomaterials are another limiting feature for 3D bioprinting, as currently there are not a large number of tested bioink compositions. The optimization or development of materials with improved properties for bioprinting is desirable. Future adoption of 3D bioprinting by non-specialists is additionally hampered by the lack of standardized printers for applications. In principle, 3D bioprinters offer reproducibility but without standardized equipment and commercially available bioinks/printing materials, inter-lab reproducibility has been limited. Furthermore, the lack of commercial sources makes it difficult for non-specialist engineers to adopt 3D bioprinting for producing *in vitro* models. Open-source 3D bioprinting, of which we are huge proponents (Kinstlinger et al., 2016; Miller, 2014; Miller et al., 2012), can boost access and standardization across lab environments, while also lowering costs and enabling greater control. The increased frequency of publications that describe 3D bioprinting methods provides the groundwork for how to build and use 3D bioprinting techniques. However, the successful adoption of these techniques into mainstream research requires transdisciplinary efforts between engineers and cancer biologists.

3D bioprinting technologies have produced amazing results for tissue engineering that could equally revolutionize our understanding of metastasis. We expect 3DP technologies to significantly expand our capability to construct complex and reproducible *in vitro* tumor models, thereby empowering cancer biologists to experience a surge of progress in elucidating the crucial yet unclear role of the TME in metastatic disease.

## References

[DMM025049C1] Abbott A. (2003). Biology's new dimension. *Nature* 424, 870-872. 10.1038/424870a 12931155

[DMM025049C2] Albrecht D. R., Underhill G. H., Wassermann T. B., Sah R. L. and Bhatia S. N. (2006). Probing the role of multicellular organization in three-dimensional microenvironments. *Nat. Methods* 3, 369-375. 10.1038/nmeth873 16628207

[DMM025049C3] Balkwill F. R. and Mantovani A. (2012). Cancer-related inflammation: common themes and therapeutic opportunities. *Semin. Cancer Biol.* 22, 33-40. 10.1016/j.semcancer.2011.12.005 22210179

[DMM025049C4] Bentz H., Schroeder J. A. and Estridge T. D. (1998). Improved local delivery of TGF-β2 by binding to injectable fibrillar collagen via difunctional polyethylene glycol. *J. Biomed. Mater. Res.* 39, 539-548. 10.1002/(SICI)1097-4636(19980315)39:4<539::AID-JBM6>3.0.CO;2-K 9492213

[DMM025049C5] Bertassoni L. E., Cecconi M., Manoharan V., Nikkhah M., Hjortnaes J., Cristino A. L., Barabaschi G., Demarchi D., Dokmeci M. R., Yang Y. et al. (2014). Hydrogel bioprinted microchannel networks for vascularization of tissue engineering constructs. *Lab. Chip* 14, 2202-2211. 10.1039/C4LC00030G 24860845 PMC4201051

[DMM025049C6] Bhattacharjee T., Zehnder S. M., Rowe K. G., Jain S., Nixon R. M., Sawyer W. G. and Angelini T. E. (2015). Writing in the Granular Gel Medium. *Sci. Adv.* 1, e1500655 10.1126/sciadv.1500655 26601274 PMC4643780

[DMM025049C7] Bissell M. J. and Hines W. C. (2011). Why don't we get more cancer? A proposed role of the microenvironment in restraining cancer progression. *Nat. Med.* 17, 320-329. 10.1038/nm.2328 21383745 PMC3569482

[DMM025049C8] Bissell M. J. and Radisky D. (2001). Putting tumours in context. *Nat. Rev. Cancer* 1, 46-54. 10.1038/35094059 11900251 PMC2975572

[DMM025049C9] Boyden S. (1962). The chemotactic effect of mixtures of antibody and antigen on polymorphonuclear leucocytes. *J. Exp. Med.* 115, 453-466. 10.1084/jem.115.3.453 13872176 PMC2137509

[DMM025049C10] Bray L. J., Binner M., Holzheu A., Friedrichs J., Freudenberg U., Hutmacher D. W. and Werner C. (2015). Multi-parametric hydrogels support 3D in vitro bioengineered microenvironment models of tumour angiogenesis. *Biomaterials* 53, 609-620. 10.1016/j.biomaterials.2015.02.124 25890757

[DMM025049C11] Burg T., Cass C. A. P., Groff R., Pepper M. and Burg K. J. L. (2010). Building off-the-shelf tissue-engineered composites. *Philos. Trans. A Math. Phys. Eng. Sci.* 368, 1839-1862. 10.1098/rsta.2010.0002 20308106

[DMM025049C12] Bussard K. M., Boulanger C. A., Booth B. W., Bruno R. D. and Smith G. H. (2010). Reprogramming human cancer cells in the mouse mammary gland. *Cancer Res.* 70, 6336-6343. 10.1158/0008-5472.CAN-10-0591 20647316 PMC3494489

[DMM025049C13] Cameron M. D., Schmidt E. E., Kerkvliet N., Nadkarni K. V., Morris V. L., Groom A. C., Chambers A. F. and MacDonald I. C. (2000). Temporal progression of metastasis in lung: cell survival, dormancy, and location dependence of metastatic inefficiency. *Cancer Res.* 60, 2541-2546. 10811137

[DMM025049C14] Carmeliet P. and Jain R. K. (2000). Angiogenesis in cancer and other diseases. *Nature* 407, 249-257. 10.1038/35025220 11001068

[DMM025049C15] Chaffer C. L. and Weinberg R. A. (2011). A perspective on cancer cell metastasis. *Science* 331, 1559-1564. 10.1126/science.1203543 21436443

[DMM025049C16] Chambers A. F., Groom A. C. and MacDonald I. C. (2002). Metastasis: dissemination and growth of cancer cells in metastatic sites. *Nat. Rev. Cancer* 2, 563-572. 10.1038/nrc865 12154349

[DMM025049C17] Chaudhuri O., Gu L., Darnell M., Klumpers D., Bencherif S. A., Weaver J. C., Huebsch N. and Mooney D. J. (2015). Substrate stress relaxation regulates cell spreading. *Nat. Commun.* 6, 6365 10.1038/ncomms7365 PMC4518451 25695512

[DMM025049C18] Colina M., Duocastella M., Fernández-Pradas J. M., Serra P. and Morenza J. L. (2006). Laser-induced forward transfer of liquids: study of the droplet ejection process. *J. Appl. Phys.* 99, 084909 10.1063/1.2191569

[DMM025049C19] Condeelis J. and Pollard J. W. (2006). Macrophages: obligate partners for tumor cell migration, invasion, and metastasis. *Cell* 124, 263-266. 10.1016/j.cell.2006.01.007 16439202

[DMM025049C20] Coussens L. M. and Werb Z. (2002). Inflammation and cancer. *Nature* 420, 860-867. 10.1038/nature01322 12490959 PMC2803035

[DMM025049C21] Coussens L. M., Coussens L. M., Zitvogel L. and Palucka A. K. (2013). Neutralizing tumor-promoting chronic inflammation: a magic bullet? *Science* 286, 286-291. 10.1126/science.1232227 PMC3591506 23329041

[DMM025049C22] Dankbar B. (2000). Vascular endothelial growth factor and interleukin-6 in paracrine tumor-stromal cell interactions in multiple myeloma. *Blood* 95, 2630-2636. 10753844

[DMM025049C23] DeForest C. A. and Anseth K. S. (2011). Cytocompatible click-based hydrogels with dynamically tunable properties through orthogonal photoconjugation and photocleavage reactions. *Nat. Chem.* 3, 925-931. 10.1038/nchem.1174 22109271 PMC3229165

[DMM025049C24] DeForest C. A. and Anseth K. S. (2012). Photoreversible patterning of biomolecules within click-based hydrogels. *Angew. Chemie Int. Ed.* 51, 1816-1819. 10.1002/anie.201106463 PMC3430005 22162285

[DMM025049C25] DeForest C. A., Sims E. A. and Anseth K. S. (2010). Peptide-functionalized click hydrogels with independently tunable mechanics and chemical functionality for 3D cell culture. *Chem. Mater.* 22, 4783-4790. 10.1021/cm101391y 20842213 PMC2937999

[DMM025049C26] DeLong S. A., Moon J. J. and West J. L. (2005). Covalently immobilized gradients of bFGF on hydrogel scaffolds for directed cell migration. *Biomaterials* 26, 3227-3234. 10.1016/j.biomaterials.2004.09.021 15603817

[DMM025049C27] Derby B. (2008). Bioprinting: inkjet printing proteins and hybrid cell-containing materials and structures. *J. Mater. Chem.* 18, 5717 10.1039/b807560c

[DMM025049C28] Dickhut A., Dexheimer V., Martin K., Lauinger R., Heisel C. and Richter W. (2010). Chondrogenesis of human mesenchymal stem cells by local transforming growth factor-beta delivery in a biphasic resorbable carrier. *Tissue Eng Part A* 16, 453-464. 10.1089/ten.tea.2009.0168 19705961

[DMM025049C29] Dolberg D. S. and Bissell M. J. (1984). Inability of Rous sarcoma virus to cause sarcomas in the avian embryo. *Nature* 309, 552-556. 10.1038/309552a0 6203040

[DMM025049C30] Eccles S. A. (2005). Targeting key steps in metastatic tumour progression. *Curr. Opin. Genet. Dev.* 15, 77-86. 10.1016/j.gde.2004.12.001 15661537

[DMM025049C31] Elomaa L., Pan C.-C., Shanjani Y., Malkovskiy A., Seppälä J. V. and Yang Y. (2015). Three-dimensional fabrication of cell-laden biodegradable poly(ethylene glycol-co-depsipeptide) hydrogels by visible light stereolithography. *J. Mater. Chem. B* 3, 8348-8358. 10.1039/C5TB01468A PMC5650242 29057076

[DMM025049C32] Engler A. J., Sen S., Sweeney H. L. and Discher D. E. (2006). Matrix elasticity directs stem cell lineage specification. *Cell* 126, 677-689. 10.1016/j.cell.2006.06.044 16923388

[DMM025049C33] Folkman J. (2002). Role of angiogenesis in tumor growth and metastasis. *Semin. Oncol.* 29, 15-18. 10.1053/sonc.2002.37263 12516034

[DMM025049C34] Friedl P. and Wolf K. (2003). Tumour-cell invasion and migration: diversity and escape mechanisms. *Nat. Rev. Cancer* 3, 362-374. 10.1038/nrc1075 12724734

[DMM025049C35] Gattazzo F., Urciuolo A. and Bonaldo P. (2014). Extracellular matrix: a dynamic microenvironment for stem cell niche. *Biochim. Biophys. Acta Gen. Subj.* 1840, 2506-2519. 10.1016/j.bbagen.2014.01.010 PMC4081568 24418517

[DMM025049C36] Giannelli G., Falk-Marzillier J., Schiraldi O., Stetler-Stevenson W. G. and Quaranta V. (1997). Induction of cell migration by matrix metalloprotease-2 cleavage of laminin-5. *Science* 277, 225-228. 10.1126/science.277.5323.225 9211848

[DMM025049C37] Gill B. J. and West J. L. (2014). Modeling the tumor extracellular matrix: tissue engineering tools repurposed towards new frontiers in cancer biology. *J. Biomech.* 47, 1969-1978. 10.1016/j.jbiomech.2013.09.029 24300038

[DMM025049C38] Gill B. J., Gibbons D. L., Roudsari L. C., Saik J. E., Rizvi Z. H., Roybal J. D., Kurie J. M. and West J. L. (2012). A synthetic matrix with independently tunable biochemistry and mechanical properties to study epithelial morphogenesis and EMT in a lung adenocarcinoma model. *Cancer Res.* 72, 6013-6023. 10.1158/0008-5472.CAN-12-0895 22952217 PMC3632398

[DMM025049C39] Greaves M. and Maley C. C. (2012). Clonal evolution in cancer. *Nature* 481, 306-313. 10.1038/nature10762 22258609 PMC3367003

[DMM025049C40] Griffith L. G. and Swartz M. A. (2006). Capturing complex 3D tissue physiology in vitro. *Nat. Rev. Mol. Cell Biol.* 7, 211-224. 10.1038/nrm1858 16496023

[DMM025049C41] Grivennikov S. I., Greten F. R. and Karin M. (2010). Immunity, inflammation, and cancer. *Cell* 140, 883-899. 10.1016/j.cell.2010.01.025 20303878 PMC2866629

[DMM025049C42] Grolman J. M., Zhang D., Smith A. M., Moore J. S. and Kilian K. A. (2015). Rapid 3D extrusion of synthetic tumor microenvironments. *Adv. Mater.* 27, 5512-5517. 10.1002/adma.201501729 26283579 PMC4745120

[DMM025049C43] Grove C. S. and Vassiliou G. S. (2014). Acute myeloid leukaemia: a paradigm for the clonal evolution of cancer? *Dis. Model. Mech.* 7, 941-951. 10.1242/dmm.015974 25056697 PMC4107323

[DMM025049C44] Gruene M., Deiwick A., Koch L., Schlie S., Unger C., Hofmann N., Bernemann I., Glasmacher B. and Chichkov B. (2011). Laser printing of stem cells for biofabrication of scaffold-free autologous grafts. *Tissue Eng. Part C Methods* 17, 79-87. 10.1089/ten.tec.2010.0359 20673023

[DMM025049C45] Guillotin B., Souquet A., Catros S., Duocastella M., Pippenger B., Bellance S., Bareille R., Rémy M., Bordenave L., Amédée J. et al. (2010). Laser assisted bioprinting of engineered tissue with high cell density and microscale organization. *Biomaterials* 31, 7250-7256. 10.1016/j.biomaterials.2010.05.055 20580082

[DMM025049C46] Gurkan U. A., Fan Y., Xu F., Erkmen B., Urkac E. S., Parlakgul G., Bernstein J., Xing W., Boyden E. S. and Demirci U. (2013). Simple precision creation of digitally specified, spatially heterogeneous, engineered tissue architectures. *Adv. Mater.* 25, 1192-1198. 10.1002/adma.201203261 23192949 PMC3842103

[DMM025049C47] Gurkan U. A., El Assal R., Yildiz S. E., Sung Y., Trachtenberg A. J., Kuo W. P. and Demirci U. (2014). Engineering anisotropic biomimetic fibrocartilage microenvironment by bioprinting mesenchymal stem cells in nanoliter gel droplets. *Mol. Pharm.* 11, 2151-2159. 10.1021/mp400573g 24495169 PMC4096228

[DMM025049C48] Guvendiren M. and Burdick J. A. (2013). Engineering synthetic hydrogel microenvironments to instruct stem cells. *Curr. Opin. Biotechnol.* 24, 841-846. 10.1016/j.copbio.2013.03.009 23545441 PMC3783596

[DMM025049C49] Haeger A., Wolf K., Zegers M. M. and Friedl P. (2015). Collective cell migration: guidance principles and hierarchies. *Trends Cell Biol.* 25, 556-566. 10.1016/j.tcb.2015.06.003 26137890

[DMM025049C50] Hanahan D. and Coussens L. M. (2012). Accessories to the crime: functions of cells recruited to the tumor microenvironment. *Cancer Cell* 21, 309-322. 10.1016/j.ccr.2012.02.022 22439926

[DMM025049C51] Hanahan D. and Weinberg R. A. (2011). Hallmarks of cancer: the next generation. *Cell* 144, 646-674. 10.1016/j.cell.2011.02.013 21376230

[DMM025049C52] Hersel U., Dahmen C. and Kessler H. (2003). RGD modified polymers: biomaterials for stimulated cell adhesion and beyond. *Biomaterials* 24, 4385-4415. 10.1016/S0142-9612(03)00343-0 12922151

[DMM025049C53] Hinton T. J., Jallerat Q., Palchesko R. N., Park J. H., Grodzicki M. S., Shue H.-J., Ramadan M. H., Hudson A. R. and Feinberg A. W. (2015). Three-dimensional printing of complex biological structures by freeform reversible embedding of suspended hydrogels. *Sci. Adv.* 1, e1500758 10.1126/sciadv.1500758 26601312 PMC4646826

[DMM025049C54] Hribar K. C., Soman P., Warner J., Chung P. and Chen S. (2014). Light-assisted direct-write of 3D functional biomaterials. *Lab Chip* 14, 268-275. 10.1039/C3LC50634G 24257507

[DMM025049C55] Hubbell J. A. (2008). Cellular matrices: physiology in microfluidics. *Nat. Mater.* 7, 609-610. 10.1038/nmat2238 18654581

[DMM025049C56] Hull C. W. (1986). Apparatus for production of three-dimensional objects by stereolithography. US Patent 4,575,330 1-16.

[DMM025049C57] Hutmacher D. W. (2010). Biomaterials offer cancer research the third dimension. *Nat. Mater.* 9, 90-93. 10.1038/nmat2619 20094076

[DMM025049C58] Hynes R. O. (2009). The extracellular matrix: not just pretty fibrils. *Science* 326, 1216-1219. 10.1126/science.1176009 19965464 PMC3536535

[DMM025049C59] Iyengar P., Espina V., Williams T. W., Lin Y., Berry D., Jelicks L. A., Lee H., Temple K., Graves R., Pollard J. et al. (2005). Adipocyte-derived collagen VI affects early mammary tumor progression in vivo, demonstrating a critical interaction in the tumor/stroma microenvironment. *J. Clin. Invest.* 115, 1163-1176. 10.1172/JCI23424 15841211 PMC1077173

[DMM025049C60] Jain R. K. (2013). Normalizing tumor microenvironment to treat cancer: bench to bedside to biomarkers. *J. Clin. Oncol.* 31, 2205-2218. 10.1200/JCO.2012.46.3653 23669226 PMC3731977

[DMM025049C61] Kaemmerer E., Melchels F. P. W., Holzapfel B. M., Meckel T., Hutmacher D. W. and Loessner D. (2014). Gelatine methacrylamide-based hydrogels: an alternative three-dimensional cancer cell culture system. *Acta Biomater.* 10, 2551-2562. 10.1016/j.actbio.2014.02.035 24590158

[DMM025049C62] Kalluri R. and Zeisberg M. (2006). Fibroblasts in cancer. *Nat. Rev. Cancer* 6, 392-401. 10.1038/nrc1877 16572188

[DMM025049C63] Kang H.-W., Lee S. J., Ko I. K., Kengla C., Yoo J. J. and Atala A. (2016). A 3D bioprinting system to produce human-scale tissue constructs with structural integrity. *Nat. Biotechnol.* 34, 312-319. 10.1038/nbt.3413 26878319

[DMM025049C64] Karagiannis G. S., Poutahidis T., Erdman S. E., Kirsch R., Riddell R. H. and Diamandis E. P. (2012). Cancer-associated fibroblasts drive the progression of metastasis through both paracrine and mechanical pressure on cancer tissue. *Mol. Cancer Res.* 10, 1403-1418. 10.1158/1541-7786.MCR-12-0307 23024188 PMC4399759

[DMM025049C65] Kim S., Kim H. J. and Jeon N. L. (2010). Biological applications of microfluidic gradient devices. *Integr. Biol.* 2, 584-603. 10.1039/c0ib00055h 20957276

[DMM025049C66] Kinstlinger I. S., Bastian A., Paulsen S. J., Hwang D. H., Ta A. H., Yalacki D. R., Schmidt T. and Miller J. S. (2016). Open-Source Selective Laser Sintering (OpenSLS) of nylon and biocompatible polycaprolactone. *PLoS ONE* 11, e0147399 10.1371/journal.pone.0147399 26841023 PMC4739701

[DMM025049C67] Kleinman H. K. and Martin G. R. (2005). Matrigel: basement membrane matrix with biological activity. *Semin. Cancer Biol.* 15, 378-386. 10.1016/j.semcancer.2005.05.004 15975825

[DMM025049C68] Knowlton S., Onal S., Yu C. H., Zhao J. J. and Tasoglu S. (2015). Bioprinting for cancer research. *Trends Biotechnol.* 33, 504-513. 10.1016/j.tibtech.2015.06.007 26216543

[DMM025049C69] Kolesky D. B., Truby R. L., Gladman A. S., Busbee T. A., Homan K. A. and Lewis J. A. (2014). 3D bioprinting of vascularized, heterogeneous cell-laden tissue constructs. *Adv. Mater.* 26, 3124-3130. 10.1002/adma.201305506 24550124

[DMM025049C70] LaFratta C. N., Li L. and Fourkas J. T. (2006). Soft-lithographic replication of 3D microstructures with closed loops. *Proc. Natl. Acad. Sci. USA* 103, 8589-8594. 10.1073/pnas.0603247103 16720698 PMC1464799

[DMM025049C71] Landskron G., De la Fuente M., Thuwajit P., Thuwajit C. and Hermoso M. A. (2014). Chronic inflammation and cytokines in the tumor microenvironment. *J. Immunol. Res.* 2014, 149185 10.1155/2014/149185 24901008 PMC4036716

[DMM025049C72] Levental K. R., Yu H., Kass L., Lakins J. N., Egeblad M., Erler J. T., Fong S. F. T., Csiszar K., Giaccia A., Weninger W. et al. (2009). Matrix crosslinking forces tumor progression by enhancing integrin signaling. *Cell* 139, 891-906. 10.1016/j.cell.2009.10.027 19931152 PMC2788004

[DMM025049C73] Li L. and Fourkas J. T. (2007). Multiphoton polymerization. *Mater. Today* 10, 30-37. 10.1016/S1369-7021(07)70130-X

[DMM025049C74] Li C., Faulkner-Jones A., Dun A. R., Jin J., Chen P., Xing Y., Yang Z., Li Z., Shu W., Liu D. et al. (2015). Rapid formation of a supramolecular polypeptide-DNA hydrogel for in situ three-dimensional multilayer bioprinting. *Angew. Chemie Int. Ed.* 54, 3957-3961. 10.1002/anie.201411383 25656851

[DMM025049C75] Liu J., Tan Y., Zhang H., Zhang Y., Xu P., Chen J., Poh Y.-C., Tang K., Wang N. and Huang B. (2012). Soft fibrin gels promote selection and growth of tumorigenic cells. *Nat. Mater.* 11, 734-741. 10.1038/nmat3361 22751180 PMC3405191

[DMM025049C76] Loessner D., Rizzi S. C., Stok K. S., Fuehrmann T., Hollier B., Magdolen V., Hutmacher D. W. and Clements J. A. (2013). A bioengineered 3D ovarian cancer model for the assessment of peptidase-mediated enhancement of spheroid growth and intraperitoneal spread. *Biomaterials* 34, 7389-7400. 10.1016/j.biomaterials.2013.06.009 23827191

[DMM025049C77] Loessner D., Meinert C., Kaemmerer E., Martine L. C., Yue K., Levett P. A., Klein T. J., Melchels F. P. W., Khademhosseini A. and Hutmacher D. W. (2016). Functionalization, preparation and use of cell-laden gelatin methacryloyl-based hydrogels as modular tissue culture platforms. *Nat. Protoc.* 11, 727-746. 10.1038/nprot.2016.037 26985572

[DMM025049C78] Luo Y. and Shoichet M. S. (2004). A photolabile hydrogel for guided three-dimensional cell growth and migration. *Nat. Mater.* 3, 249-253. 10.1038/nmat1092 15034559

[DMM025049C79] Lutolf M. P. and Hubbell J. A. (2005). Synthetic biomaterials as instructive extracellular microenvironments for morphogenesis in tissue engineering. *Nat. Biotechnol.* 23, 47-55. 10.1038/nbt1055 15637621

[DMM025049C80] Luzzi K. J., MacDonald I. C., Schmidt E. E., Kerkvliet N., Morris V. L., Chambers A. F. and Groom A. C. (1998). Multistep nature of metastatic inefficiency: dormancy of solitary cells after successful extravasation and limited survival of early micrometastases. *Am. J. Pathol.* 153, 865-873. 10.1016/S0002-9440(10)65628-3 9736035 PMC1853000

[DMM025049C81] Maffini M. V., Soto A. M., Calabro J. M., Ucci A. A. and Sonnenschein C. (2004). The stroma as a crucial target in rat mammary gland carcinogenesis. *J. Cell Sci.* 117, 1495-1502. 10.1242/jcs.01000 14996910

[DMM025049C82] Mann B. K., Schmedlen R. H. and West J. L. (2001). Tethered-TGF-β increases extracellular matrix production of vascular smooth muscle cells. *Biomaterials* 22, 439-444. 10.1016/S0142-9612(00)00196-4 11214754

[DMM025049C83] Massagué J. and Obenauf A. C. (2016). Metastatic colonization by circulating tumour cells. *Nature* 529, 298-306. 10.1038/nature17038 26791720 PMC5029466

[DMM025049C84] McDonald D. M. and Baluk P. (2002). Significance of blood vessel leakiness in cancer. *Cancer Res.* 62, 5381-5385. 12235011

[DMM025049C85] Melchels F. P. W., Feijen J. and Grijpma D. W. (2010). A review on stereolithography and its applications in biomedical engineering. *Biomaterials* 31, 6121-6130. 10.1016/j.biomaterials.2010.04.050 20478613

[DMM025049C86] Merlo L. M. F., Pepper J. W., Reid B. J. and Maley C. C. (2006). Cancer as an evolutionary and ecological process. *Nat. Rev. Cancer* 6, 924-935. 10.1038/nrc2013 17109012

[DMM025049C87] Miller J. S. (2014). The billion cell construct: will three-dimensional printing get us there? *PLoS Biol.* 12, 1-9. 10.1371/journal.pbio.1001882 PMC4061004 24937565

[DMM025049C88] Miller J. S., Béthencourt M. I., Hahn M., Lee T. R. and West J. L. (2006). Laser-scanning lithography (LSL) for the soft lithographic patterning of cell-adhesive self-assembled monolayers. *Biotechnol. Bioeng.* 93, 1060-1068. 10.1002/bit.20809 16444742

[DMM025049C89] Miller J. S., Stevens K. R., Yang M. T., Baker B. M., Nguyen D.-H. T., Cohen D. M., Toro E., Chen A. A., Galie P. A., Yu X. et al. (2012). Rapid casting of patterned vascular networks for perfusable engineered three-dimensional tissues. *Nat. Mater.* 11, 768-774. 10.1038/nmat3357 22751181 PMC3586565

[DMM025049C90] Mosadegh B., Lockett M. R., Minn K. T., Simon K. A., Gilbert K., Hillier S., Newsome D., Li H., Hall A. B., Boucher D. M. et al. (2015). A paper-based invasion assay: assessing chemotaxis of cancer cells in gradients of oxygen. *Biomaterials* 52, 262-271. 10.1016/j.biomaterials.2015.02.012 25818432

[DMM025049C91] Mosiewicz K. A., Kolb L., van der Vlies A. J. and Lutolf M. P. (2014). Microscale patterning of hydrogel stiffness through light-triggered uncaging of thiols. *Biomater. Sci.* 2, 1640-1651. 10.1039/C4BM00262H 32481945

[DMM025049C92] Mouw J. K., Ou G. and Weaver V. M. (2014). Extracellular matrix assembly: a multiscale deconstruction. *Nat. Rev. Mol. Cell Biol.* 15, 771-785. 10.1038/nrm3902 25370693 PMC4682873

[DMM025049C93] Murphy S. V. and Atala A. (2014). 3D bioprinting of tissues and organs. *Nat. Biotechnol.* 32, 773-785. 10.1038/nbt.2958 25093879

[DMM025049C94] Neiman J. A. S., Raman R., Chan V., Rhoads M. G., Raredon M. S. B., Velazquez J. J., Dyer R. L., Bashir R., Hammond P. T. and Griffith L. G. (2015). Photopatterning of hydrogel scaffolds coupled to filter materials using stereolithography for perfused 3D culture of hepatocytes. *Biotechnol. Bioeng.* 112, 777-787. 10.1002/bit.25494 25384798 PMC4624302

[DMM025049C95] Nowell P. C. (1976). The clonal evolution of tumor cell populations. *Science* 194, 23-28. 10.1126/science.959840 959840

[DMM025049C96] Orimo A. and Weinberg R. A. (2006). Stromal fibroblasts in cancer: a novel tumor-promoting cell type. *Cell Cycle* 5, 1597-1601. 10.4161/cc.5.15.3112 16880743

[DMM025049C97] Ovsianikov A., Gruene M., Pflaum M., Koch L., Maiorana F., Wilhelmi M., Haverich A. and Chichkov B. (2010). Laser printing of cells into 3D scaffolds. *Biofabrication* 2, 014104 10.1088/1758-5082/2/1/014104 20811119

[DMM025049C98] Pàez-Ribes M., Allen E., Hudock J., Takeda T., Okuyama H., Viñals F., Inoue M., Bergers G., Hanahan D. and Casanovas O. (2009). Antiangiogenic therapy elicits malignant progression of tumors to increased local invasion and distant metastasis. *Cancer Cell* 15, 220-231. 10.1016/j.ccr.2009.01.027 19249680 PMC2874829

[DMM025049C99] Paget S. (1989). The distribution of secondary growths in cancer of the breast. *Cancer Metastasis Rev.* 8, 98-101. 2673568

[DMM025049C100] Papetti M. and Herman I. M. (2002). Mechanisms of normal and tumor-derived angiogenesis. *Am. J. Physiol. Cell Physiol.* 282, C947-C970. 10.1152/ajpcell.00389.2001 11940508

[DMM025049C101] Paszek M. J., Zahir N., Johnson K. R., Lakins J. N., Rozenberg G. I., Gefen A., Reinhart-King C. A., Margulies S. S., Dembo M., Boettiger D. et al. (2005). Tensional homeostasis and the malignant phenotype. *Cancer Cell* 8, 241-254. 10.1016/j.ccr.2005.08.010 16169468

[DMM025049C102] Pati F., Jang J., Ha D.-H., Won Kim S., Rhie J.-W., Shim J.-H., Kim D.-H. and Cho D.-W. (2014). Printing three-dimensional tissue analogues with decellularized extracellular matrix bioink. *Nat. Commun.* 5, 1-11. 10.1038/ncomms4935 PMC4059935 24887553

[DMM025049C103] Pati F., Jang J., Lee J. W. and Cho D.-W. (2015). Chapter 7 - Extrusion bioprinting. In *Essentials of 3D Biofabrication and Translation* (ed. Atala A. and Yoo J. J.), pp 123-152. Boston: Academic Press 10.1016/B978-0-12-800972-7.00007-4

[DMM025049C104] Petersen O. W., Rønnov-Jessen L., Howlett A. R. and Bissell M. J. (1992). Interaction with basement membrane serves to rapidly distinguish growth and differentiation pattern of normal and malignant human breast epithelial cells. *Proc. Natl. Acad. Sci. USA* 89, 9064-9068. 10.1073/pnas.89.19.9064 1384042 PMC50065

[DMM025049C105] Pittenger M. F. (1999). Multilineage potential of adult human mesenchymal stem cells. *Science* 284, 143-147. 10.1126/science.284.5411.143 10102814

[DMM025049C106] Polacheck W. J., Charest J. L. and Kamm R. D. (2011). Interstitial flow influences direction of tumor cell migration through competing mechanisms. *Proc. Natl. Acad. Sci. USA* 108, 11115-11120. 10.1073/pnas.1103581108 21690404 PMC3131352

[DMM025049C107] Provenzano P. P., Inman D. R., Eliceiri K. W., Knittel J. G., Yan L., Rueden C. T., White J. G. and Keely P. J. (2008). Collagen density promotes mammary tumor initiation and progression. *BMC Med.* 6, 11 10.1186/1741-7015-6-11 18442412 PMC2386807

[DMM025049C108] Quail D. F. and Joyce J. A. (2013). Microenvironmental regulation of tumor progression and metastasis. *Nat. Med.* 19, 1423-1437. 10.1038/nm.3394 24202395 PMC3954707

[DMM025049C109] Raeber G. P., Lutolf M. P. and Hubbell J. A. (2005). Molecularly engineered PEG hydrogels: a novel model system for proteolytically mediated cell migration. *Biophys. J.* 89, 1374-1388. 10.1529/biophysj.104.050682 15923238 PMC1366622

[DMM025049C110] Reynolds A. R., Hart I. R., Watson A. R., Welti J. C., Silva R. G., Robinson S. D., Da Violante G., Gourlaouen M., Salih M., Jones M. C. et al. (2009). Stimulation of tumor growth and angiogenesis by low concentrations of RGD-mimetic integrin inhibitors. *Nat. Med.* 15, 392-400. 10.1038/nm.1941 19305413

[DMM025049C111] Riching K. M., Cox B. L., Salick M. R., Pehlke C., Riching A. S., Ponik S. M., Bass B. R., Crone W. C., Jiang Y., Weaver A. M. et al. (2015). 3D collagen alignment limits protrusions to enhance breast cancer cell persistence. *Biophys. J.* 107, 2546-2558. 10.1016/j.bpj.2014.10.035 PMC4255204 25468334

[DMM025049C112] Roskoski R. Jr (2007). Vascular endothelial growth factor (VEGF) signaling in tumor progression. *Crit. Rev. Oncol. Hematol.* 62, 179-213. 10.1016/j.critrevonc.2007.01.006 17324579

[DMM025049C113] Rozario T. and DeSimone D. W. (2010). The extracellular matrix in development and morphogenesis: a dynamic view. *Dev. Biol.* 341, 126-140. 10.1016/j.ydbio.2009.10.026 19854168 PMC2854274

[DMM025049C114] Ruoslahti E. (1996). Rgd and other recognition sequences for integrins. *Annu. Rev. Cell Dev. Biol* 12, 697-715. 10.1146/annurev.cellbio.12.1.697 8970741

[DMM025049C115] Schuessler T. K., Chan X. Y., Chen H. J., Ji K., Park K. M., Roshan-Ghias A., Sethi P., Thakur A., Tian X., Villasante A. et al. (2014). Biomimetic tissue-engineered systems for advancing cancer research: NCI Strategic Workshop report. *Cancer Res.* 74, 5359-5363. 10.1158/0008-5472.CAN-14-1706 25095784 PMC4184963

[DMM025049C116] Sears N. A., Seshadri D. R., Dhavalikar P. S. and Cosgriff-Hernandez E. (2016). A review of 3D printing of tissue engineering. *Tissue Eng. Part B. Rev.* 22, 298-310. 10.1089/ten.teb.2015.0464 26857350

[DMM025049C117] Shen Y.-I., Abaci H. E., Krupski Y., Weng L.-C., Burdick J. A. and Gerecht S. (2014). Hyaluronic acid hydrogel stiffness and oxygen tension affect cancer cell fate and endothelial sprouting. *Biomater. Sci.* 2, 655-665. 10.1039/c3bm60274e 24748963 PMC3987918

[DMM025049C118] Shim J.-H., Lee J.-S., Kim J. Y. and Cho D.-W. (2012). Bioprinting of a mechanically enhanced three-dimensional dual cell-laden construct for osteochondral tissue engineering using a multi-head tissue/organ building system. *J. Micromech. Microeng.* 22, 085014 10.1088/0960-1317/22/8/085014

[DMM025049C119] Shirazi S. F. S., Gharehkhani S., Mehrali M., Yarmand H., Metselaar H. S. C., Adib Kadri N. and Osman N. A. A. (2015). A review on powder-based additive manufacturing for tissue engineering: selective laser sintering and inkjet 3D printing. *Sci. Technol. Adv. Mater.* 16, 033502 10.1088/1468-6996/16/3/033502 27877783 PMC5099820

[DMM025049C120] Siegel R. L., Miller K. D. and Jemal A. (2015). Cancer statistics, 2015. *CA Cancer J. Clin.* 65, 5-29. 10.3322/caac.21254 25559415

[DMM025049C121] Sionkowska A. (2011). Current research on the blends of natural and synthetic polymers as new biomaterials: review. *Prog. Polym. Sci.* 36, 1254-1276. 10.1016/j.progpolymsci.2011.05.003

[DMM025049C122] Sooppan R., Paulsen S. J., Han J., Ta A. H., Dinh P., Gaffey A. C., Venkataraman C., Trubelja A., Hung G., Miller J. S. et al. (2016). In vivo anastomosis and perfusion of a three-dimensionally-printed construct containing microchannel networks. *Tissue Eng. Part C. Methods* 22, 1-7. 10.1089/ten.tec.2015.0239 26414863 PMC4722541

[DMM025049C123] Strieter R. M., Belperio J. A., Phillips R. J. and Keane M. P. (2004). CXC chemokines in angiogenesis of cancer. *Semin. Cancer Biol.* 14, 195-200. 10.1016/j.semcancer.2003.10.006 15246055

[DMM025049C124] Sydney Gladman A., Matsumoto E. A., Nuzzo R. G., Mahadevan L. and Lewis J. A. (2016). Biomimetic 4D printing. *Nat. Mater.* 15, 413-418. 10.1038/nmat4544 26808461

[DMM025049C125] Tasoglu S. and Demirci U. (2013). Bioprinting for stem cell research. *Trends Biotechnol* 31, 10-19. 10.1016/j.tibtech.2012.10.005 23260439 PMC3534918

[DMM025049C126] Tibbitt M. W. and Anseth K. S. (2009). Hydrogels as extracellular matrix mimics for 3D cell culture. *Biotechnol. Bioeng.* 103, 655-663. 10.1002/bit.22361 19472329 PMC2997742

[DMM025049C127] Torre L. A., Bray F., Siegel R. L., Ferlay J., Lortet-tieulent J. and Jemal A. (2015). Global Cancer Statistics, 2012. *CA A Cancer J. Clin.* 65, 87-108. 10.3322/caac.21262 25651787

[DMM025049C128] Verbridge S. S., Chakrabarti A., DelNero P., Kwee B., Varner J. D., Stroock A. D. and Fischbach C. (2013). Physicochemical regulation of endothelial sprouting in a 3D microfluidic angiogenesis model. *J. Biomed. Mater. Res. Part A* 101, 2948-2956. 10.1002/jbm.a.34587 PMC3776016 23559519

[DMM025049C129] Wagenseil J. E. and Mecham R. P. (2009). Vascular extracellular matrix and arterial mechanics. *Physiol. Rev.* 89, 957-989. 10.1152/physrev.00041.2008 19584318 PMC2775470

[DMM025049C130] Watt F. M. and Huck W. T. S. (2013). Role of the extracellular matrix in regulating stem cell fate. *Nat. Rev. Mol. Cell Biol.* 14, 467-473. 10.1038/nrm3620 23839578

[DMM025049C131] Weaver V. M., Petersen O. W., Wang F., Larabell C. A., Briand P., Damsky C. and Bissell M. J. (1997). Reversion of the malignant phenotype of human breast cells in three-dimensional culture and in vivo by integrin blocking antibodies. *J. Cell Biol.* 137, 231-245. 10.1083/jcb.137.1.231 9105051 PMC2139858

[DMM025049C132] Wu W., Deconinck A. and Lewis J. A. (2011). Omnidirectional printing of 3D microvascular networks. *Adv. Mater.* 23, H178-H183. 10.1002/adma.201004625 21438034

[DMM025049C133] Wyckoff J., Wang W., Lin E. Y., Wang Y., Pixley F., Stanley E. R., Graf T., Pollard J. W., Segall J. and Condeelis J. (2004). A paracrine loop between tumor cells and macrophages is required for tumor cell migration in mammary tumors. *Cancer Res.* 64, 7022-7029. 10.1158/0008-5472.CAN-04-1449 15466195

[DMM025049C134] Xia Y. and Whitesides G. M. (1998). Soft lithography. *Annu. Rev. Mater. Sci.* 28, 153-184. 10.1146/annurev.matsci.28.1.153

[DMM025049C135] Xing J.-F., Zheng M.-L. and Duan X.-M. (2015). Two-photon polymerization microfabrication of hydrogels: an advanced 3D printing technology for tissue engineering and drug delivery. *Chem. Soc. Rev.* 44, 5031-5039. 10.1039/C5CS00278H 25992492

[DMM025049C136] Yamada K. M. and Cukierman E. (2007). Modeling tissue morphogenesis and cancer in 3D. *Cell* 130, 601-610. 10.1016/j.cell.2007.08.006 17719539

[DMM025049C137] Yamaguchi H., Wyckoff J. and Condeelis J. (2005). Cell migration in tumors. *Curr. Opin. Cell Biol.* 17, 559-564. 10.1016/j.ceb.2005.08.002 16098726

[DMM025049C138] Young E. W. K. and Beebe D. J. (2010). Fundamentals of microfluidic cell culture in controlled microenvironments. *Chem. Soc. Rev.* 39, 1036-1048. 10.1039/b909900j 20179823 PMC2967183

[DMM025049C139] Yu Q. and Stamenkovic I. (2000). Cell surface-localized metalloproteinase-9 proteolyically activates TGF-beta and promotes tumour invasion and angiogenesis. *Genes Dev.* 14, 163-176. 10652271 PMC316345

[DMM025049C140] Yu H., Mouw J. K. and Weaver V. M. (2011). Forcing form and function: biomechanical regulation of tumor evolution. *Trends Cell Biol.* 21, 47-56. 10.1016/j.tcb.2010.08.015 20870407 PMC3014395

[DMM025049C141] Zaman M. H., Trapani L. M., Sieminski A. L., Siemeski A., MacKellar D., Gong H., Kamm R. D., Wells A., Lauffenburger D. A. and Matsudaira P. (2006). Migration of tumor cells in 3D matrices is governed by matrix stiffness along with cell-matrix adhesion and proteolysis. *Proc. Natl. Acad. Sci. USA* 103, 10889-10894. 10.1073/pnas.0604460103 16832052 PMC1544144

[DMM025049C142] Zein I., Hutmacher D. W., Tan K. C. and Teoh S. H. (2002). Fused deposition modeling of novel scaffold architectures for tissue engineering applications. *Biomaterials* 23, 1169-1185. 10.1016/S0142-9612(01)00232-0 11791921

[DMM025049C143] Zervantonakis I. K., Hughes-Alford S. K., Charest J. L., Condeelis J. S., Gertler F. B. and Kamm R. D. (2012). Three-dimensional microfluidic model for tumor cell intravasation and endothelial barrier function. *Proc. Natl. Acad. Sci. USA* 109, 13515-13520. 10.1073/pnas.1210182109 22869695 PMC3427099

[DMM025049C144] Zhu J. (2010). Bioactive modification of poly(ethylene glycol) hydrogels for tissue engineering. *Biomaterials* 31, 4639-4656. 10.1016/j.biomaterials.2010.02.044 20303169 PMC2907908

